# Comparison of thoracic computed tomography and surgical reports in dogs and cats

**DOI:** 10.3389/fvets.2025.1566436

**Published:** 2025-07-21

**Authors:** Maja Brložnik, Martin Immler, Maria Laura Prüllage, Olivia Mariel Grünzweil, Manolis Lyrakis, Eberhard Ludewig, Sibylle Maria Kneissl

**Affiliations:** ^1^Clinical Department for Small Animals and Horses, Diagnostic Imaging, University of Veterinary Medicine, Vienna (Vetmeduni), Vienna, Austria; ^2^Clinical Department for Small Animals and Horses, Small Animal Surgery, University of Veterinary Medicine, Vienna (Vetmeduni), Vienna, Austria; ^3^Department of Biological Sciences and Pathobiology, Platform for Bioinformatics and Biostatistics, University of Veterinary Medicine, Vienna (Vetmeduni), Vienna, Austria

**Keywords:** dog, cat, computed tomography, cognitive error, perception error, discrepancy

## Abstract

**Introduction:**

This retrospective study compared computed tomography (CT) and surgical reports in 41 dogs and 23 cats undergoing thoracic surgery (50 thoracotomies, 14 thoracoscopies).

**Objectives:**

The objective of this study was to evaluate the agreement between imaging and surgical findings in dogs and cats undergoing thoracic surgery and to access sensitivity of imaging for major surgical finding.

**Methods:**

Patients were included if they had an in-house CT study performed within 8 days prior to surgery, had a finalized CT report available before surgery, and if the corresponding surgical report was sufficiently detailed to allow meaningful comparison with CT findings. Imaging and surgical findings were extracted and categorized as complete agreement, partial agreement (regarding type, number, or site of lesion), no agreement, or equivocal. Short-term outcome (discharged or deceased) was recorded.

**Results:**

Agreement between primary imaging and surgical findings was achieved in 55 of 64 patients (86%): 33 dogs (33/41; 80%) and 22 cats (22/23; 96%). No agreement was found in 6 of 64 patients (9%): bullae were not detected in 3 dogs and 1 cat, a foreign body was missed in 1 dog, and pericarditis was missed in 1 dog. Partial agreement was found in one patient with several bullae (2%). Based on the available data, 2 of 64 patients could not be classified (3%). Surgical reports documented that the most common lesions were pleural effusion (12 dogs, 15 cats) and pulmonary mass/nodule (14 dogs, 5 cats). Fifty-two patients were discharged, while twelve (12/64; 19%) died before discharge (six patients died and six were euthanized). Significant association between categories of pathology and agreement was observed (*p* < 0.001). The categories of pathology with the highest number of cases (lung mass (*n* = 19) and pleural effusion (*n* = 27)) showed only complete agreement between primary imaging and surgical findings. Differences in agreement were associated with perception (*n* = 4), cognitive error (*n* = 2) and discrepancy (*n* = 1).

**Conclusion:**

Small and camouflaged pathologies, such as low-contrast foreign bodies and ruptured bullae in the atelectatic lung, were most frequently missed or wrongly interpreted in CT reports.

## 1 Introduction

In thoracic surgery, an accurate preoperative diagnosis is critical for determining the appropriate surgical approach and improving outcomes. Computed tomography (CT) is an indispensable imaging tool for evaluating thoracic diseases in small animals, offering detailed visualization of thoracic structures. However, despite its diagnostic value, CT has limitations and may yield false results due to limitations inherent in the method. Disagreement between preoperative imaging and intraoperative findings remain a challenge in clinical practice and have been reported in several studies (1–3). For example, clinical history has been shown to significantly impact the interpretation and diagnostic accuracy of thoracic radiographs (4). Also, studies have highlighted disagreement in imaging modalities, such as radiographs failing to detect pulmonary nodules later identified on CT (5). Additionally, certain imaging techniques may be more effective for specific diagnostic challenges; for example, ultrasonography has been shown to aid in detecting foreign bodies, especially during preoperative evaluations (6), whereas CT is superior in identifying subtle or complex lesions, such as wooden foreign bodies or thoracic lymphatic structures (7, 8). Operator experience and training can improve interpretation of images from modalities such as ultrasonography and radiography (9, 10). These findings emphasize the need to improve diagnostic accuracy in veterinary medicine.

Research in human medicine suggests that disagreement between imaging and surgical findings may result from perceptual errors (missed abnormalities) and cognitive errors (wrong interpretation of detected findings) (11, 12). Perceptual errors occur when subtle findings are overlooked, such as small pulmonary nodules mistaken for artifacts or subtle pneumothorax misinterpreted as normal pleural anatomy (5, 13). Cognitive errors arise when cognitive biases influence radiologists’ interpretations (12, 14). Examples of cognitive factors include subsequent search miss (15), originally known as satisfaction of search (16), where the detection of one abnormality leads to a premature conclusion of the search, causing additional abnormalities to be overlooked; and inattentional blindness, where an individual fails to perceive an unexpected stimulus in plain sight because their attention is focused on another task or object (13, 17). Other cognitive biases, such as anchoring bias, where initial impressions disproportionately influence subsequent interpretations, or framing effects, where contextual information skews decision-making, may also play a role (11, 18).

Understanding the causes of errors is essential for minimizing their occurrence. Also, identifying the types of lesions that are more likely to be missed or misinterpreted during CT evaluations is crucial for improving diagnostic performance in veterinary practice. The aim of this study was to evaluate the agreement between imaging and surgical findings in dogs and cats undergoing thoracic surgery and to access sensitivity of imaging for major surgical findings. We hypothesized that agreement between imaging and surgical reports would be good and that specific lesion types—particularly smaller or camouflaged pathologies—would be more prone to error.

## 2 Materials and methods

Medical records of dogs and cats that underwent thoracic surgery (thoracotomy or thoracoscopy) from 2014 to 2024 at Vetmeduni were retrieved. Patients were included if they had an in-house CT study performed within 8 days prior to surgery, had a finalized CT report available before surgery, and if the corresponding surgical report was sufficiently detailed to allow meaningful comparison with CT findings. Intraoperative findings served as the gold standard for evaluating the sensitivity of CT reports. The goal was to determine whether the findings from the CT reports were consistent or inconsistent with the surgical findings. Cases were excluded if surgical exploration was not considered an adequate gold standard. For example, in a case of pericardial neoplasia, thoracoscopy and pericardiectomy were performed from the contralateral hemithorax, which did not allow adequate exploration of the affected side.

Surgical findings were classified as follows: (A) thoracic trauma; (B) lung mass; (C) mass other than the lung; (D) pulmonary bullae or blebs causing pneumothorax; (E) non-traumatic or neoplastic pleural effusion, and (F) other single or rare events such as a foreign body or vascular ring anomaly.

Primary lesions were defined as the main surgical finding, while secondary lesions were categorized as any other associated surgical finding. For example, in an animal with a pulmonary mass and pleural effusion, the pulmonary mass was classified as primary and the pleural effusion as secondary. Histological results were considered if the classification of imaging or surgical findings was inconclusive. Any findings on CT or at surgery that were considered by the surgeon to be unrelated to the primary lesion were also recorded. In cases of uncertainty regarding whether the imaging and surgical findings referred to the same lesion or when descriptions were ambiguous, a consensus was reached among four radiologists (M.B., M.P., O.G., S.K.). Surgical outcomes (discharged, died, or euthanized) were also recorded.

Agreement was classified as follows: no agreement (main surgical findings were not documented in the imaging reports), agreement (main surgical findings were described in the imaging reports), partial agreement (main findings were noted but errors in site or other details occurred), or not defined based on the information available.

Perceptual errors were defined as findings that were described by the surgeon but not mentioned in the radiology report (false negatives), or findings described by the radiologist but not confirmed by the surgeon (false positives). Cognitive errors referred to imaging findings that were correctly described but misinterpreted in the context of clinical reasoning—thus representing true positives with incorrect conclusions. Error classification was based solely on written reports; CT images were not re-evaluated. For instance, if a pulmonary bulla was not mentioned by the radiologist but identified by the surgeon, it was classified as a perceptual error. Conversely, if a mass was accurately described by the radiologist but misinterpreted as a neoplasm, the error was classified as cognitive. The term ‘discrepancy’ was used to describe a mismatch that could be explained by reasonable differences of opinion between colleagues or by knowledge gained through the surgical report or patient outcome (13).

Statistical analysis was performed with R version 4.4.1 (R: A Language and Environment for Statistical Computing version 4.4.1, R Core Team [2024])^1^. Associations between agreement (i.e., agreement vs. partial agreement & disagreement) and diagnosis, procedure or outcome were evaluated via separate Fisher’s exact tests for count data (function fisher.test). Two cases, which could not be classified, were excluded. Multiple Fisher’s exact tests were corrected for multiple testing using the Bonferroni–Holm method (function p.adjust). Significance was declared at 5% cut-off after multiple testing correction. The sensitivity of imaging for major surgical finding was calculated as the ratio of true positives to the sum of true positives and false negatives.

## 3 Results

The characteristics of the 64 included cases (50 thoracotomies and 14 thoracoscopies in 41 dogs and 23 cats) are shown in Table 1.

**Table 1:** Surgical procedure, age, sex, category of pathology, and outcome of included 64 cases with thoracic surgery.

Criterion	Attributes	All (*n* = 64)	Dogs (*n* = 41)	Cats (*n* = 23)
Procedure	Thoracotomy	50 (78%)	30 (73%)	20 (87%)
	Thoracoscopy	14 (22%)	11 (27%)	3 (13%)
Average age (years)		7.3	7.5	6.9
Sex	Male neutered	24 (37%)	8 (19%)	16 (70%)
	Male intact	12 (19%)	11 (27%)	1 (4%)
	Female neutered	23 (36%)	18 (44%)	5 (22%)
	Female intact	5 (8%)	4 (10%)	1 (4%)
Category of pathology	Thoracic trauma	2 (3%)	2 (5%)	0
	Lung mass	19 (30%)	14 (34%)	5 (22%)
	Mass other than lung	3 (5%)	1 (2%)	2 (9%)
	Bullae or blebs causing pneumothorax	7 (11%)	6 (15%)	1 (4%)
	Non-traumatic or neoplastic pleural effusion	27 (42%)	12 (29%)	15 (65%)
	Other single or rare events	6 (9%)	6 (15%)	0
Outcome	Died/euthanized	12 (19%)	8 (20%)	4 (17%)
	Discharged	52 (81%)	33 (80%)	19 (83%)

Initially, 166 cases of thoracic surgeries were retrieved: 114 thoracotomies (76 dogs and 38 cats) and 52 thoracoscopies (42 dogs and 10 cats). A total of 102 patients were excluded.

Thoracic CT scans reported in this study were acquired using either a 128-slice scanner (Somatom X.cite, Siemens Healthineers, Erlangen, Germany) or, for cases prior to June 2021, a 16-slice scanner (Somatom Emotion 16, Siemens Healthineers, Erlangen, Germany). Imaging parameters included a tube voltage of 110–130 kVp, a tube current–time product of 100–350 mAs, a pitch of 0.8–1, a reconstructed slice thickness of 0.75–8 mm, and a rotation time of 0.3–1 s. Patients were positioned in sternal or dorsal recumbency, and images were acquired in the transverse plane using reconstruction algorithms for lung, bone, and soft tissue. Anesthetic management was individualized and determined by the attending anesthesiologist. In most cases, animals were fasted for 6 to 8 h, preoxygenated with 100% oxygen, and induced with propofol. Intubation was followed by maintenance with isoflurane. Breath-holding techniques were employed during image acquisition when feasible, but the type of breath-hold (neutral or positive pressure) varied between patients depending on anesthetic management and clinical stability.

Surgical reports revealed that the most common lesions were pleural effusion in 27 cases (27/64; 42%; 12 dogs, 15 cats), pulmonary mass or nodule in 19 cases (19/64; 30%; 14 dogs, 5 cats), and pulmonary bullae or blebs causing pneumothorax in 7 cases (7/64; 6 dogs, 1 cat) (Table 1). Pneumothorax was present in all cases involving bullae or blebs. However, as pneumothorax is inevitably induced during thoracic surgery, it cannot be diagnosed intraoperatively and was therefore not analyzed further.

Fifty-two patients (52/64; 81%) were discharged, while twelve patients (12/64; 19%) died before discharge (six patients died and six were euthanized). The average time until death was 2.7 days, with a standard deviation of 3.1 days.

Agreement between imaging and surgical findings was achieved in 55 of 64 patients (86%): 33 dogs (33/41; 80%) and 22 cats (22/23; 96%) (Table 2). No agreement was observed in 6 of 64 patients (9%): In four cases (3 dogs, 1 cat), bullae that were identified during surgery were not described in the CT report. In one dog, a foreign body identified surgically was not reported in the CT report. In another dog, pericarditis diagnosed during surgery had not been mentioned in the CT report.

**Table 2:** Agreement between imaging and surgery in dogs and cats undergoing thoracic surgery (64 cases).

Agreement	All (*n* = 64)	Dogs (*n* = 41)	Cats (*n* = 23)
Agreement	55 (85.9%)	33 (80.5%)	22 (95.7%)
Partial agreement	1 (1.6%)	1 (12.2%)	0
No agreement	6 (9.4%)	5 (12.2%)	1 (4.3%)
Not to be defined	2 (3.1%)	2 (4.9%)	0

Partial agreement was observed in one patient (2%) with multiple pulmonary bullae; the CT report and surgical findings differed in the number or location of lesions described. Based on the available data, 2 of 64 patients, both having pleural effusion, could not be classified (3%). In one case, the primary finding was a pericardial mass, which was histologically confirmed as inflammatory but was not identified as such by neither the radiologist nor the surgeon. In another case, the primary diagnosis was a histologically confirmed serositis, which likewise was not documented by neither the radiologist nor the surgeon. Additionally, enlarged lymph nodes noted by the radiologist were not confirmed in the surgical report.

Mismatches in agreement were attributed to perceptual errors (n = 4), cognitive errors (n = 2), or discrepancies (*n* = 1) (Table 3).

**Table 3:** Confusion matrix comparing imaging and surgical findings (59 cases).

		Confirmed in surgery	
		Surgery positive	Surgery negative
Presumed in radiology	Imaging positive	55 (TP)	0 (FP)
	Imaging negative	4* (FN)	0 (TN)

Five cases with cognitive errors (*n* = 2^#^), discrepancy (*n* = 1^§^) or insufficient detail (*n* = 2) were excluded.

FN, false negative; FP, false positive; TN, true negative; TP, true positive. *Two cases with bullae/blebs, one case with a foreign body and one case with a pericarditis were missed by the radiologist (4x perceptual error). ^#^One foreign body was presumed to be a ruptured pulmonary bulla, while in the other case one pulmonary bulla was presumed to be a foreign body (2x cognitive error). Perceptual and cognitive errors resulted in 6 cases with ‘no agreement’ in Table 2. ^§^One case with pulmonary bullae could not be evaluated because of an atelectatic lung lobe, whereas the leakage test performed during surgery was positive (1x discrepancy). This case was categorized as ‘partial agreement’ in Table 2 because only the site of the problem was correctly described by radiologist.

Significant association between the categories of pathology and agreement was observed (*p* < 0.001). The categories of pathology with the highest number of cases – lung mass (n = 19) and pleural effusion (*n* = 27) – showed only complete agreement between primary imaging and surgical findings (Table 4). However, no significant association was found between agreement and surgical procedure or outcome (*p* > 0.05). The sensitivity of CT for detection of lesions was 93%.

**Table 4:** Agreement between imaging and surgery of all cases in each category of pathology (64 cases).

Category of pathology	No agreement	Partial agreement	Agreement	Not to be defined
Thoracic trauma	1	0	1	0
Lung mass	0	0	19	0
Mass other than the lung	1	0	2	0
Pulmonary bullae or blebs causing pneumothorax	3	1	3	0
Non-traumatic or neoplastic pleural effusion	0	0	25	2
Other single or rare events	1	0	5	0
Sum	**6**	**1**	**55**	**2**

A significant association between the categories of pathology and agreement was observed (*p* < 0.001).

## 4 Discussion

This study evaluated the agreement between CT reports and surgical findings in identifying thoracic abnormalities in dogs and cats undergoing thoracotomy or thoracoscopy. The overall agreement between preoperative imaging and surgical findings (86%), along with a sensitivity of 93%, highlights the clinical utility of CT in the preoperative assessment of thoracic surgical cases. Notably, CT demonstrated perfect concordance with surgical findings (100%) in cases involving pleural effusion and pulmonary masses, underscoring its high reliability in identifying these common thoracic conditions. Prior studies have emphasized the superior spatial resolution and diagnostic performance of CT relative to radiography, particularly in the detection of pulmonary nodules and thoracic masses (2, 3, 5, 19–24), as well as in the delineation of pleural effusion and its associated pathologies (25–27).

Despite these strengths, false negatives were observed in 6% of cases, reflecting not only the limitations of CT scans in identifying certain pathologies but also instances where CT-documented lesions were not confirmed intraoperatively. Challenges were particularly noted with bullae and blebs (agreement of only 43%) and small foreign bodies, consistent with previous reports highlighting the difficulty of visualizing small, camouflaged pathologies (8, 13, 28). Foreign bodies, especially radiolucent materials, can be obscured by the thoracic environment (6, 7, 29). These objects can blend into the surrounding tissue or be overlooked due to their radiographic appearance (30). The poor agreement observed for pulmonary bullae and blebs in this study aligns with the challenges described in detecting subtle emphysematous changes or small peripheral lesions (31–33). Although effective in many cases, it can be difficult for a helical CT scan to detect these subtle abnormalities due to their variable appearance. Previous research has noted that bullae may appear as subpleural, semicircular hyperlucent structures with partially imperceptible walls that are easily overlooked during imaging evaluation (32, 33). These findings emphasize the importance of using high-resolution thin-slice CT (33) and dedicated training to improve detection in these conditions. Furthermore, a recent study demonstrated that acquiring CT scans in both sternal and dorsal recumbency can significantly improve the identification of pulmonary bullae in dogs with spontaneous pneumothorax. This is likely due to changes in aeration and lesion conspicuity caused by positional changes (32). This technique could help to overcome limitations related to lesion location and subtle wall margins and should be considered when bullae are suspected.

Two cases that met the study’s inclusion criteria were not classified, as inflammation was only confirmed by histopathology. The limitations of CT in detecting inflammatory or infectious conditions in dogs are well documented. For example, in a retrospective study of 52 dogs with pleural effusion showed considerable overlap in CT features between inflammatory and malignant conditions, suggesting that CT alone may be insufficient for accurate differentiation (34).

Another challenge was the absence of a definitive conclusion in some radiology reports, which was particularly problematic in cases involving multiple findings. This may be due to the complexity of the findings, which challenge a clear conclusion. Radiologists also avoid synthesis by recommending further diagnostics, or adopting a non-decisive defensive approach. Nevertheless, current reporting guidelines encourage the inclusion of a diagnostic conclusion (35). This practice is now being promoted within our group to improve the clinical utility of radiology reports.

Perceptual errors—false negative CT findings—were found in four cases, including missed bullae, a foreign body, and pericarditis. Such errors are often linked to fatigue, time pressure, or cognitive overload, and may involve subsequent search miss (12, 15, 17, 18). Two cases showed cognitive errors regarding the main surgical finding. One foreign body was presumed to be a ruptured pulmonary bulla, while in the other case one pulmonary bulla was presumed to be a foreign body. A cognitive (“thinking”) error – a true positive CT finding but incorrect interpretation – is considered a misjudgment or an erroneous conclusion, often resulting from the brain’s tendency to simplify complex information. This filtering process enables rapid information processing but can also lead to inaccurate assessments. In film reading, cognitive errors lead to inaccurate clinical reasoning; they occur in 2–20% of radiologic findings (13) and arise when changes are seen on an image but misinterpreted in the medical context. They are associated with a lack of knowledge, incorrect data acquisition or processing, or deficits in metacognition (36). The probability of a cognitive error tends to increase with professional experience, as increasing diagnostic certainty may be accompanied by reduced critical reflection (10). One case showed a discrepancy. Discrepancies do not necessarily constitute errors (13, 18), but rather explain the limitation of tests such as CT in detecting pulmonary bullae when the pulmonary lobe is atelectatic (Table 3).

These findings highlight the need for strategies to reduce diagnostic errors, particularly in complex cases where subtle abnormalities or biases may lead to disagreement. Although strategies such as structured training and standardized reporting protocols have been shown to reduce errors (11, 37), evidence from radiology suggests that double reading—where two radiologists independently or collaboratively review imaging studies—is the most effective way to minimize diagnostic errors or discrepancies (13, 14). Double reading has been shown to reduce both perceptual and cognitive errors (10, 38), as a second reader can identify abnormalities or biases missed by the first. Incorporating double reading or second-reader systems into routine practice may enhance diagnostic accuracy in veterinary imaging, particularly in complex or ambiguous cases. Additionally, artificial intelligence tools, although not a replacement for radiologists, could serve as a supplementary resource to highlight areas of potential diagnostic concern (37, 39). The impact of diagnostic errors or discrepancies on clinical decision-making was beyond the scope of this study and was therefore not assessed systematically. Based on the available clinical information, it is likely that the observed diagnostic errors or discrepancies had little influence on clinical outcomes. In most cases, surgical intervention had already been planned before imaging took place, with CT primarily being used to guide or refine the surgical approach.

There are several limitations to this study that should be acknowledged. Firstly, the small sample size may limit the study’s statistical power and generalizability. Nevertheless, we observed a clear association between pathology categories and agreement, indicating greater robustness than initially expected. Secondly, the retrospective design relied on the accuracy and completeness of imaging and surgical records, which may have introduced selection bias. Cases with missing data or delays between imaging and surgery were excluded, which may affect the representativeness of the sample. Third, small subgroup sizes, particularly for conditions such as bullae and blebs, also limited our ability to assess CT sensitivity for these lesions. At Vetmeduni, radiology reports are generated in a clinical setting by either a senior radiologist, a board-certified radiologist, or a supervised resident, using a dual reading approach. Differences in experience, interpretation, and reporting style may have contributed to errors or discrepancies. Some missed findings were could have been genuinely difficult to detect. Technical limitations such as inadequate breath-holding, small lesion size, low contrast, proximity to atelectatic lung, or motion artifacts may also have contributed to errors or discrepancies. Finally, while it is feasible to categorize perceptual and cognitive errors based on reports, it is essential to acknowledge the limitations of this approach. Retrospective reviews may be influenced by hindsight bias. Some perceptual errors might not have been identified, if the missed finding was not documented in the surgery report. Without images, it is challenging to assess the complexity of subtle findings, which can influence or lead to inaccurate error classification.

This study confirms the value of CT for preoperative planning in thoracic surgery, particularly for conditions such as pleural effusion and pulmonary masses, where agreement with surgical findings was consistently good. In contrast, poor agreement for subtle pathologies such as bullae and radiolucent foreign bodies highlights the need for high-resolution thin-section imaging and dedicated training. These lesions may be more susceptible to misinterpretation or oversight, highlighting the importance of addressing human factors, such as perceptual and cognitive errors. While this study did not evaluate specific imaging strategies, the findings highlight the need for approaches aimed at reducing diagnostic error. Previous studies have proposed various strategies for improving lesion detection, such as high-resolution thin-slice imaging (33), an adequate breath-hold technique (5), dual-recumbency CT (32) and structured reporting systems (35). Dual reading and supplemental tools, such as artificial intelligence, have also been suggested as a means of reducing human error (38, 39). While not all methods may be feasible in every clinical setting, they offer valuable ways to improve diagnostic accuracy. Further studies directly comparing imaging and surgical findings are essential to better understand diagnostic errors or discrepancies.

## Data Availability

The original contributions presented in the study are included in the article/supplementary material, further inquiries can be directed to the corresponding author.

## Footnotes

^1^ https://www.R-project.org

## Author’s note

Part of this study has been presented as a poster at EVDI Annual Conference, SEP 18-21, 2024, Athens, Greece.
